# Gelatin Hydrolysate Hybrid Nanoparticles as Soft Edible Pickering Stabilizers for Oil-In-Water Emulsions

**DOI:** 10.3390/molecules25020393

**Published:** 2020-01-17

**Authors:** Zhongyao Du, Pengjie Wang

**Affiliations:** Beijing Advanced Innovation Center for Food Nutrition and Human Health, College of Food Science & Nutritional Engineering, China Agricultural University, Beijing 10036, China; duzhongyao@cau.edu.cn

**Keywords:** edible pickering stabilizer, emulsifying property, gelatin hydrolysate hybrid, soft gel particles

## Abstract

The aim of this study was to fabricate edible gelatin enzymic digest (GED) based gel particles that can stabilize oil-in-water (O/W) microemulsions. The gel particles were generated by covalent crosslinking, with genipin, the individual protein molecules within tannic acid-induced gelatin hydrolysate (GED-TA) particles. The ability of the genipin-treated GED-TA (GP-GED-TA) to stabilize emulsions was evaluated by Turbiscan analysis and droplet-size changes. For comparison, gelatin hydrolysate (GE) and tannic acid-induced gelatin hydrolysate particles (GED-TA) were used as controls. The mean diameters of GED, GED-TA, and GP-GED-TA particles were 0.68 ± 0.1 nm, 66.2 ± 8.4 nm, and 66.9 ± 7.2 nm, respectively. Nanomechanic analysis using atomic force microscopy(AFM) indicated the average Young’s modulu of the GP-GED-TA particles was 760.8 ± 112.0 Mpa, indicating the GP-GED-TA were soft particles. The Turbiscan stability indexes (lower values indicate a more stable emulsion) of the emulsions stabilized with GED, GED-TA, and GP-GED-TA, after storage for three days, were 28.6 ± 1.5, 19.3 ± 4.8, and 4.4 ± 1.3, respectively. After one, or 60 days of storage, the volume-weighted mean diameters (D_[4,3]_) of oil droplets stabilized by GP-GED-TA were 1.19 ± 0.11 μm and 1.18 ± 0.1 µm, respectively. The D_[4,3]_ of oil droplets stabilized by GED-TA, however, increased from 108.3 ± 5.1 μm to 164.3 ± 19.1 μm during the storage. Overall, the GP-GED-TA gel particles have considerable potential for stabilization of O/W emulsions in food products.

## 1. Introduction

Oil-in-water (O/W) emulsions are systems consisting of oil phase dispersed in aqueous phase [1]. They are used in many food products, such as salad dressings and mayonnaise [2,3]. Conventionally, O/W emulsions are prepared and stabilized by surfactants, but recent reports have focused on O/W Pickering emulsions, which are stabilized by nanoparticles [4,5,6]. Particles effectively inhibit the flocculation or coalescence of oil droplets in emulsions, by surrounding the oil droplets and keeping them apart [7,8]. In addition, the adsorption energy of a particle at the oil–water interface is much higher than its thermal energy. Therefore, particles are irreversibly adsorbed at the oil–water interface [9,10].

Particles for stabilizing Pickering emulsions can be classified as inorganic solid particles and organic particles [11,12,13,14]. Inorganic solid particles are usually not applicable in the food industry. Currently, many edible organic particles for stabilizing O/W emulsions have been developed, including starch particles [15], cellulose particles [16], chitin particles [17], and protein colloidal particles [18]. Soft protein particles have gained the most attention [19,20] because they have dangling protein chains on their surfaces that give them an enhanced ability to accumulate at the interface, similar to protein molecules. Once adsorbed onto the interface, they form a viscoelastic layer with higher mechanical strength than that of rigid particles (such as cellulose or chitin particles). Therefore, soft protein particles have better emulsifying activity and emulsion stabilization [21].

To date, different protein-based particle stabilizers have been reported, including zein [22], whey protein [18], peanut protein [23], and soybean protein [24]. However, they are usually rigid particles, because they are generally prepared by heat treatment, or antisolvent precipitation, resulting in highly crosslinked polymer-based particles [25]. Therefore, the development of edible soft particles could have wide application in food emulsion-based products.

Gelatin is an inexpensive food additive which is produced by collagen hydrolysis. Several instances of the preparation of soft gelatin Pickering particles have been reported. Tan et al. developed gelatin Pickering particles, using glutaraldehyde as a crosslinker [26]. However, glutaraldehyde is toxic which restricts its application in food emulsions. Feng et al. prepared gelatin particles by a two-step desolvation procedure, using genipin as a crosslinking agent [27]. Genipin is a natural crosslinking agent extracted from *Gardenia jasminoides* E. which has low toxicity to the human body. However, the particle diameter produced by this method was larger than 350 nm. This means that these particles could only stabilize oil droplets larger than 3.5 μm, since the diameter of particle should be at least one order of magnitude smaller than the targeted oil droplets [28].

**Figure 1:**
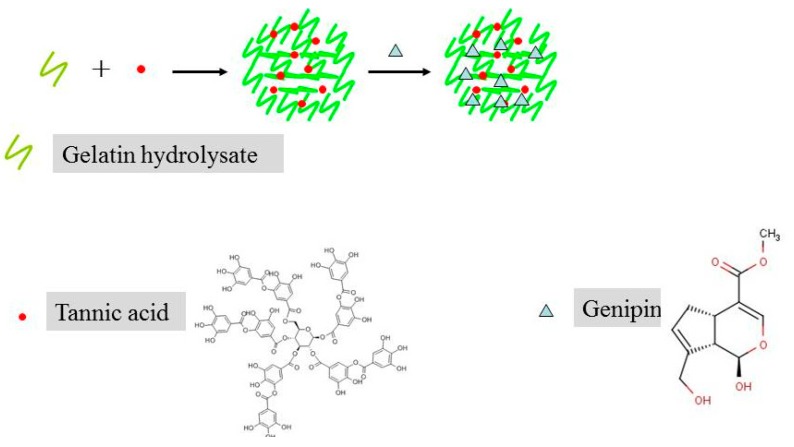
Schematic diagram of the preparation of genipin-treated tannic acid-induced gelatin hydrolysate (GP-GED-TA) gel particles.

In this study, we aimed to develop edible soft particles that could stabilize oil droplets smaller than 1.5 μm. The particles were composed of gelatin enzymatic digests (GED) and tannic acid (TA) (Figure 1), which are commercially readily available. The high binding affinity between GED and TA favors the formation of colloidal particles [29]. The formation of GED-TA particles is associated with hydrogen bonding between the hydroxyl groups in TA and the hydroxyproline residues in GED [29]. However, the GED-TA particles are noncovalent crosslinking, which could be easily dissociated at the oil–water interfaces. In this case, the introduction of covalent crosslinking agent could fabricate to form particles that cannot dissociate at the interfaces. Therefore, we used an edible covalent crosslinking agent, genipin, to further crosslink the GED-TA particles. Partly crosslinking the amino groups within the particles with genipin has generated stable Pickering stabilizers [30]. Therefore, in this study, we aimed to create edible gel particles, smaller than 150 nm, from GED and TA, and to test their ability to stabilize oil droplets in O/W emulsions.

## 2. Results

### 2.1. Characterization of Gel Particles

**Figure 2:**
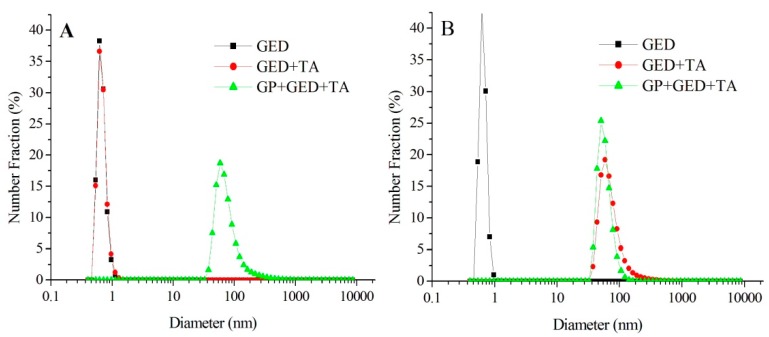
Size distribution curves, from zeta potential measurements, of particles in 8 mol L^−1^ urea solutions (**A**) or in water (**B**).

The formation of crosslinks in the genipin-treated tannic acid-induced gelatin hydrolysate (GP-GED-TA) particles was verified using a dissociation agent. In the presence of 8 mol L^−1^ urea solution, particles stabilized only by noncovalent bonds dissociate into individual molecules [31,32]. However, covalently crosslinked samples do not dissociate into individual molecules [33]. The mean diameter of GED-TA particles dispersed in water was 66.2 ± 8.4 nm (*n* = 3), but decreased to 0.69 ± 0.1 nm in the presence of 8 mol L^−1^ urea (Figure 2). The GED-TA particles clearly dissociated into individual GED molecules in urea solution, as their particle size (0.69 ± 0.1 nm vs. 0.68 ± 0.1 nm) was identical to GED in water. However, for GP-GED-TA, the particle size in urea solution was not significantly different from that in pure water (70.9 ± 12.1 nm VS. 66.9 ± 7.2 nm). This confirmed that GP-GED-TA particles could maintain their integrity and were resistant to dissociation into individual GED molecules at oil–water interfaces.

**Figure 3:**
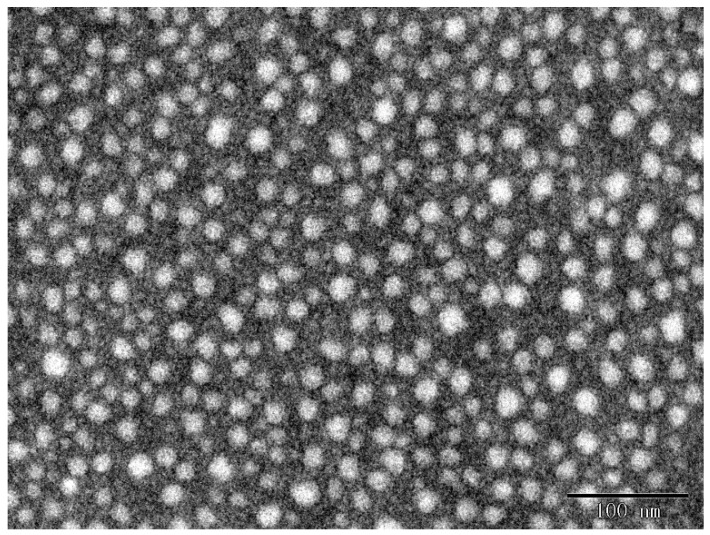
Transmission electron microscopy (TEM) images of GP-GED-TA particles. The scale bar is 100 nm.

The morphologies of the particles were observed by transmission electron microscopy (TEM) in Figure 3. It was confirmed that the particles are gspherical, with a diameter lower than the light scattering data. This is because the GP-GED-TA particles dehydrated during TEM sample preparation in in atmosphere environment.

**Figure 4:**
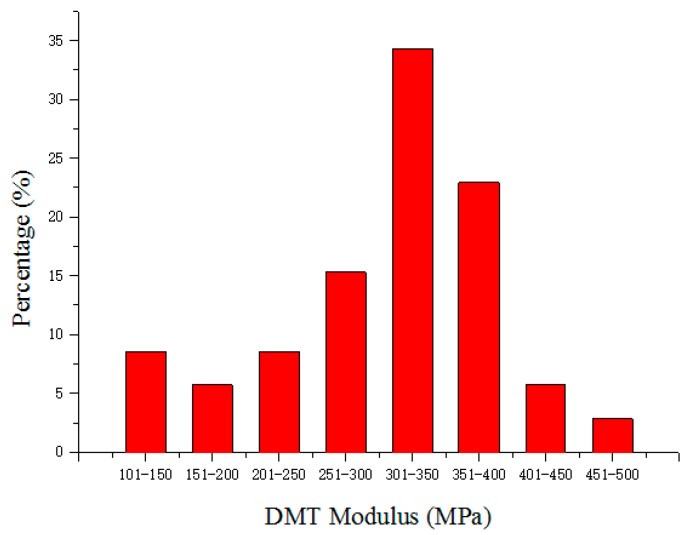
Distribution of the Young’s modulus of GP-GED-TA particles in Derjaguin-Müller-Toporov (DMT) modulus channels.

Nanomechanic analysis using peak-force quantitative nanomechanical atomic force microscopy (AFM) indicated the average Young’s modulus of the GP-GED-TA particles was 311.5 ± 80.2 MPa (*n* = 35, calculated from arithmetic mean value in Figure 4). This value was similar to the results of protein-based particles [34,35], but larger than the inorganic particles [36,37]. This indicated the GP-GED-TA particles were soft. From the AFM results, the GP-GED-TA was proven to be soft particles, which have advantages over rigid solid particles in stabilizing the oil droplets. First, it has dangling polymer chains on the surfaces which have the ability of anchoring at the interfaces. This endowed the GP-GED-TA particles higher emulsifying capacity than solid hard particles. Secondly, the particles could deform and flatten at the oil–water interface, which could, thus, form a network at the interface [12]. This favors the formation of interfacial film with higher elasticity and strength as compared with hard particles. Therefore, the covalently crosslinked GP-GED-TA particles could have better emulsifying stability than hard particles.

### 2.2. Stability of Pickering Emulsions

**Figure 5:**
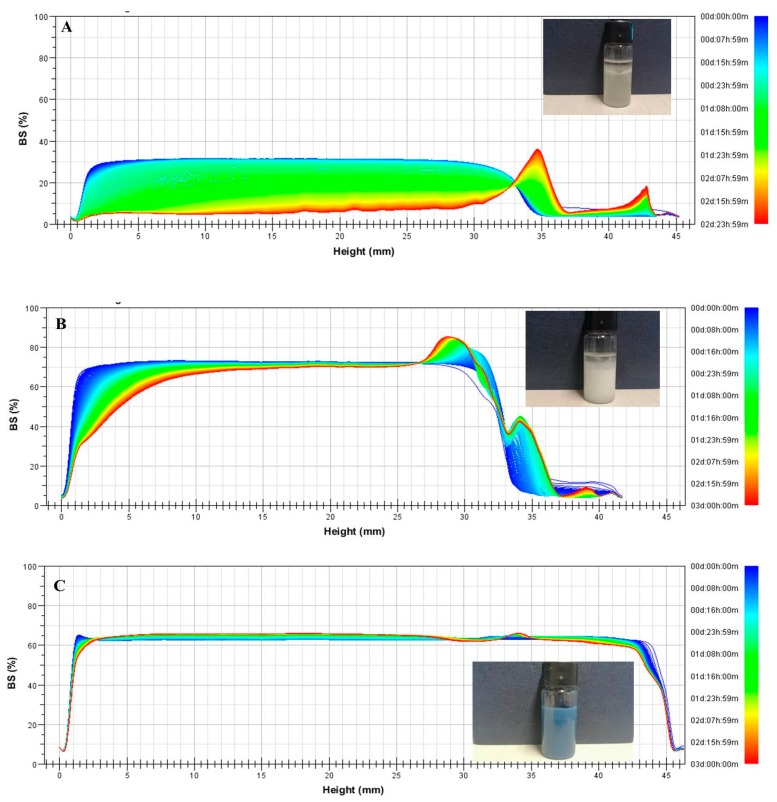
Changes in the backscattering intensity (BS) profiles of oil-in-water (O/W) emulsions generated by gelatin enzymic digest (GED) (**A**), tannic acid-induced gelatin hydrolysate (GED-TA) (**B**), and GP-GED-TA (**C**). The inserted photographs were taken 3 days after preparation of fresh emulsions.

The migration (sedimentation or creaming) and size variation (coalescence or flocculation) of particles in O/W emulsions can be easily detected by the Turbiscan (Figure 5). The backscatterings of the emulsion phase formed by GE, GE-TA, and GP-GE-TA were 3.4 ± 1.0%, 76.5 ± 4.1%, and 98.4 ± 1.3% (*n* = 3), respectively (20 min after the preparation of emulsions). This indicated that the oil droplets coalesced rapidly after the preparation of emulsions and that GED was the poorest emulsion stabilizer. This also suggested the oil droplets generated cannot be fully covered by GED-TA, while oil droplets formed by GP-GED-TA showed the highest stability against coalescence. The backscattering changes in the bottom parts (5 mm above the bottom, for instance) of the O/W emulsions stabilized by GP-GED-TA and GED-TA were 2.5 ± 0.5% and 27.3 ± 3.5%, respectively. This indicated the physical stability against coalescence, or aggregation of the oil droplets stabilized by GP-GED-TA, was much higher than those stabilized by GED-TA. Drastic changes in BS profiles were observed between about 33 to 43 mm and 27 to 42 mm, respectively (Figure 5A,B). This is because the oil droplets creamed at these positions, whereas such changes were not observed in Figure 5C. This suggested that the emulsions prepared with GP-GED-TA exhibited higher stability against creaming than those prepared with GED-TA or GED.

**Figure 6:**
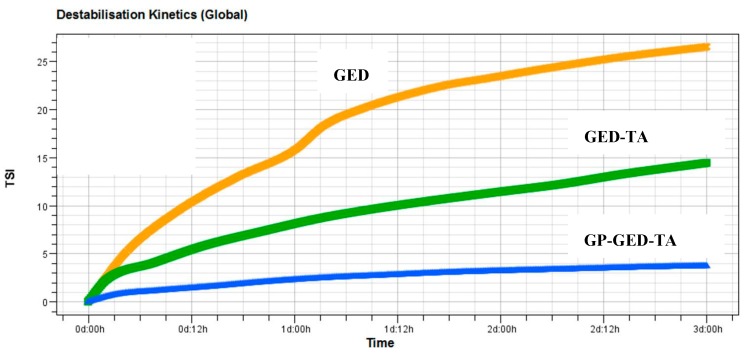
Global turbiscan stability index (TSI) of O/W emulsions generated by different stabilizing agents.

The global TSI values can be used to assess the global stability of O/W emulsions. The higher the TSI value, the less stable the emulsion. The TSI values of the three emulsions increased over time (Figure 6), which indicated the O/W emulsions destabilized during storage. However, the order of global TSI is GED (28.6 ± 1.5) > GED-TA (19.3 ± 4.8) > GP-GED-TA (4.4 ± 1.3). Therefore, the GP-GED-TA stabilized emulsion exhibited the highest stability during storage.

### 2.3. Viscosity and Size Distribution of the Emulsions

**Figure 7:**
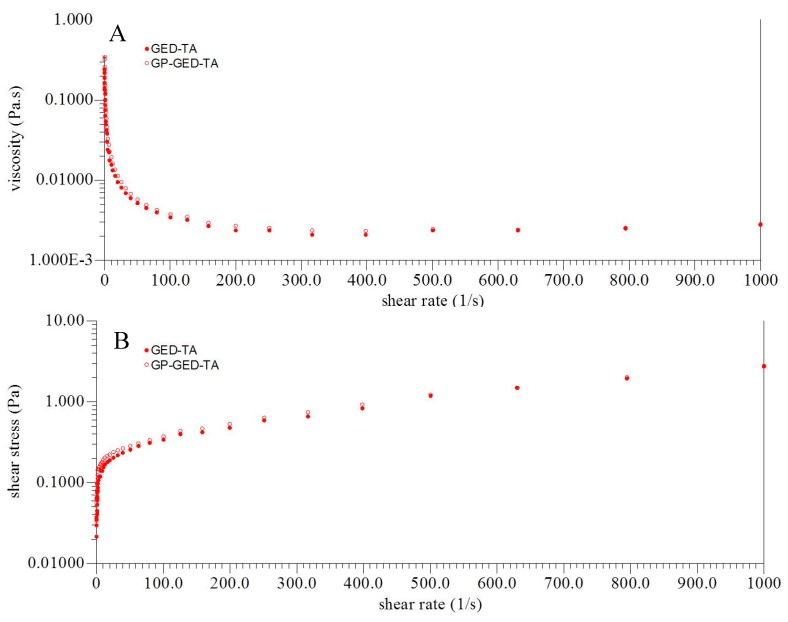
Shear-rate dependence of viscosity (**A**) and shear stress (**B**) for emulsions prepared with GED-TA or GP-GED-TA.

The migration (sedimentation or creaming) of the oil droplets is largely determined by the viscosities of the emulsions and the oil droplet sizes. No significant difference was found between the viscosities of the freshly-prepared emulsions stabilized by GED-TA and GP-GED-TA, respectively (Figure 7A). When the shear rate was 100 (1/s), the viscosities of emulsion stabilized by GED-TA or GP-GED-TA were 3.4 ± 0.1 and 3.4 ± 0.1(Pa1), respectively. This demonstrated that the higher stability of the emulsions generated by GP-GED-TA as compared with GED-TA was not caused by the viscosity difference of the emulsions. The emulsions formed by GED-TA or GP-GED-TA exhibited the shear thinning behavior at shear rates of 1 to 1000 (1/s), suggesting formation of a weak network structure in emulsion stabilized by the GED-TA or GP-GED-TA [8].

The shear stress versus shear rate data are present in Figure 7B. The data were fitted to the Herschel–Bulkley model σ = σ_0_ + K·γ^n^, as shown in Table 1. The σ is the shear stress (Pa), σ_0_ is the apparent yield stress (Pa), K is the consistency index (Pa·s^n^), γ is the shear rate (s^−1^), and n is the flow behavior index. The parameters suggested the emulsion prepared with GED-TA or GP-GED-TA exhibited shear-thinning behavior. This is due to the breaking of polymer networks during shearing, leading to the less intermolecular resistance to flow.

**Figure 8:**
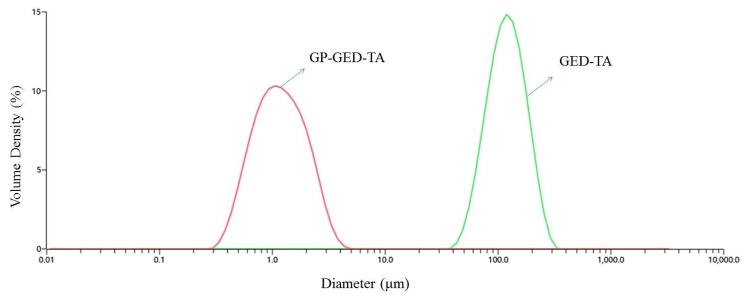
The size distribution curves of oil droplets stabilized by GP-GED-TA and GED-TA.

Oil droplets stabilized by GP-GED-TA were around two orders of magnitude smaller than those stabilized by GED-TA (Figure 8). Three hours after the preparation of emulsions, the volume-weighted mean diameters (D_[4,3]_) of oil droplets stabilized by GP-GED-TA and GED-TA were 1.19 ± 0.11 μm and 108.3 ± 5.1 μm (*n* = 3), respectively, while the corresponding polydispersity indexes (PDIs) were 1.05 ± 0.01 and 1.17 ± 0.26, respectively. After 60 days of storage, the difference was even larger, the D_[4,3]_ values were 1.18 ± 0.1 μm and 164.3 ± 19.1 μm, respectively, while the corresponding PDIs were 1.04 ± 0.02 and 1.15 ± 0.14, respectively. Therefore, the GP-GED-TA particles exhibited much higher emulsion stabilization than GED-TA.

## 3. Materials and Methods

### 3.1. Materials

GED and TA were from Sigma-Aldrich (St. Louis, MO, USA). Genipin was from Chengdu Jin Taihe Medicinal Chemistry Tech Co., Ltd. (Chengdu, Sichuan, China). Medium-chain triglycerides were bought from Beijing Keao Co., Ltd. (Beijing, China), which contained 65% caprylic acid and 35% capric acids. The melting point of MCT is −13.6 °C. All other chemical reagents used were of analytical grade and from local suppliers.

### 3.2. Preparation and Characterization of Gel Particles

GED was dispersed in deionized water (with 0.02% (*w*/*v*) sodium azide as antibacterial agent) to 2% *w*/*v*, on a magnetic stirrer at 300 rpm for 2 h at 43 °C. To prepare the GED-TA complex particles, 0.3 g/mL TA solution was added dropwise into the GED dispersions while stirring. The pH of the dispersions was kept constant (pH 7.0) with 0.1 mol L^−1^ NaOH during the addition of TA solution. The final mass ratios of TA to GED were 0 and 1:25, respectively. To crosslink the molecules in the particles, an ethanolic solution of genipin was added to the stirred GED-TA dispersions. The final mass ratio of genipin to GED in each sample was 1:10. The dispersions were stirred for 24 h at 43 °C, while the pH was maintained at 7.0 with 0.1 mol L^−1^ NaOH. The dispersions were ultrafiltered with 10 kDa membranes (UFC803096, Millipore, Burlington, USA) to remove the un-crosslinked molecules. The GP-GED-TA samples were freeze-dried to constant weight.

The GP-GED-TA dispersions were prepared by dispersing the freeze-dried GP-GED-TA samples to the concentration of 2% (*w*/*v*), on a magnetic stirrer at 500 rpm for 1 h at 25 °C. The GED dispersions were prepared by dispersing GED to the concentration of 2% (*w*/*v*). The GED-TA dispersions were prepared by adding 0.3 g/mL TA solution dropwise into the 2% GED dispersions while stirring. The final mass ratio of TA to GED was 1:25 in GED-TA dispersions. The GP-GED-TA, GED-TA, and GED dispersions contained 0.02% sodium azide. The particle size was determined with a particle analyzer (Nano ZS, Malvern, UK) at 25 °C. The samples were diluted 600 times with deionized water or 8 mol L^−1^ urea solution. The viscosity values of water and 8 mol L^−1^ urea urea solutions were 0.8872 and 1.5956 mpa s, respectively. The refractive index values of water and urea solution were 1.330 and 1.399, respectively. The refractive index values and viscosity values for both water and urea solution at given temperatures were choosed directly from the soft package of the particle analyzer. The morphologies of the particles in samples were observed by transmission electron microscopy (TEM, Tecnai-20, Philips, Eindhoven, The Netherlands) at 200 kV. Five microliters of GP-GED-TA particle dispersions (0.01%, *w*/*v*) were deposited onto a carbon-coated grid and dried in air after negative stained with 2% uranyl acetate. The elastic modulus and force maps were measured by atomic force microscopy (AFM, Dimension, Bruker, Germany) [34]. Samples were diluted 2000-fold with purified water before AFM measurement. Five microliters of the samples were spread onto a mica sheet (one centimeter in diameter) for 10 min before AFM measurement. During AFM measurement, the scanning rate and peak fore amplitude were 1.98 Hz and 150 nm, respectively. The following equation was applied to calculate a local reduced elastic modulus [34]. The Derjaguin-Müller-Toporov (DMT) model was applied to fit the data.

(1) $$F_{\mathrm{interaction}}=(4/3)E^{*}{\left(d-d_{0}\right)}^{3/2}+F_{adh}$$

where F_interaction_ represented the tip-sample force, E* was the reduced elastic modulus of the tip and the sample, R was the tip radius, *d*_0_ was the surface rest position, *d* − *d*_0_ was the depth of indentation, and *F_adh_* was the constant adhesion force during the contact.

### 3.3. Preparation and Characterization of O/W Emulsions

Medium-chain triglycerides and particle dispersions were mixed at the volume ratio of 2:3. The mixed liquids were sonicated (Scientz-IID, Ningbo, China) at 25% amplitude for 2 min (working time, 5 s and rest time, 5 s).

The stability of the emulsions was determined with a Turbiscan (Tower, Formulaction, France). Thirty minutes after preparation, the O/W emulsions were added into the Turbiscan test specific transparent tubes. The samples were scanned from bottom to top of the tubes over time with a laser (λ_air_ = 880 nm) for 72 h at 40 °C. The creaming, coalescence/flocculation of O/W emulsions, and Turbiscan stability index (TSI) were calculated from the changes in the backscattered light intensity [38]. The TSI was calculated using the following formula:

(2) $$TSI=\sqrt{\frac{\sum_{i=1}^{n}{\left(x_{i}-x_{BS}\right)}^{2}}{n-1}}$$

where *x_i_* is the backscattering for each measurement, *x_BS_* is the mean of *x_i_*, and *n* is the scan number.

The oil-droplet size of oil-in-water emulsions was measured using a Mastersizer 3000 (Malvern Instruments, Malvern, UK) after 1 and 60 days of storage at ambient temperature (~25 °C). Distilled water (25 °C) was used as the dispersion medium; the refractive index values input was 1.333. The emulsion was added into the water dropwise. When the laser intensity reached 10% to 20%, the measurement was started. The mixing speed for the dispersion medium was 2300 rpm. The average droplet size was expressed in terms of volume mean diameter D_[4,3]_ [39]. The polydispersity index (PDI) of oil droplets in emulsions was calculated using the following formula [40]:

(3) $$PDI=\frac{d_{90}-d_{10}}{d_{50}}$$

where *d*_10_, *d*_50_, and *d*_90_ represent the diameter of oil droplets at 10%, 50%, and 90% of cumulative volume.

The viscosity of the O/W emulsions (1 h after preparation) was determined using a rheometer (AR1500ex, TA Instruments, New Castle, DE, USA) with a 60 mm diameter probe [21]. The emulsions were subjected to steady shearing at 25 °C. The points per decade were 10. Three milliliters of the emulsions were placed onto the plate. The shear rate was set from 1 to 1000 s^−1^.

### 3.4. Statistical Analysis

The experiments were performed dependently at least three times. The means and standard deviations (means ± SD) were applied to express the results. Statistical differences were measured by one-way analysis of variance (ANOVA) and *p* < 0.05 was defined as significant.

## 4. Conclusions

In this study, edible gelatin enzymatic digest-based hybrid gel particles were fabricated and their ability to stabilize O/W emulsions was evaluated. The GP-GED-TA particles were stable against urea dissociation, due to the covalently crosslinking of the protein network within the TA-induced GED particles. The GP-GED-TA particle was 66.9 nm in an average hydrated diameter. The GP-GEN-TA particles displayed excellent emulsifying stability against coalescence, aggregation, and creaming (emulsions were stable for at least 60 days) as compared with O/W emulsions generated solely with GED-TA particles. It should also be noted that the softness and size of the gel particles and the pH and ionic concentration of the aqueous phase could also influence the stability of emulsions. We plan to evaluate the effects of these factors in our future work.

## Figures and Tables

**Table 1:** Shear-rate dependence of viscosity for O/W emulsion data fitting by the Herschel–Bulkley model.

Group	σ_0_ (Pa)	K (Pa·s^n^, 10^−4^)	*n*
GED-TA	0.09 ± 0.06	8.55 ± 2.24	1.17 ± 0.02
GP-GED-TA	0.11 ± 0.00	6.26 ± 1.69	1.19 ± 0.04

*n* = 3 and no significant difference was observed within the same column (*p* > 0.05).
